# Primary Progressive Multiple Sclerosis in a Portuguese Patient With Neurofibromatosis Type 1

**DOI:** 10.7759/cureus.20561

**Published:** 2021-12-21

**Authors:** Inês Carvalho, Miguel Quintas-Neves, Joana Pinto, Ana Filipa Santos, João Pereira

**Affiliations:** 1 Neurology, Hospital of Braga, Braga, PRT; 2 Neuroradiology, Hospital of Braga, Braga, PRT

**Keywords:** genetic variant, autoimmune, primary progressive, multiple sclerosis, neurofibromatosis type 1

## Abstract

Neurofibromatosis type 1 (NF1) is a frequent genetic neurocutaneous syndrome and multiple sclerosis (MS) is an acquired demyelinating disease of the central nervous system. The association of both these diseases is rare. In this case report, we describe a 25-year-old man with gait impairment, upper limbs tremor, slurred speech, and urinary symptoms in the form of urinary urgency and incontinence. These symptoms started a year earlier and had a progressive course. Examination revealed scattered café-au-lait spots, right ptosis, bilateral horizontal and vertical nystagmus, mild dysarthria, quadriparesis with generalized hyperreflexia and bilateral Babinski signs, upper limb tremor, bilateral proprioceptive errors, bilateral appendicular dysmetria, and severe gait ataxia. Brain MRI showed lesions involving the deep and subcortical white matter, as well as thalami, with no enhancement after administration of gadolinium, suggestive of focal areas of signal intensity (FASI) in the setting of NF1. There were also oval lesions in the periventricular white matter, perpendicular to the ventricles and involving the corpus callosum, which were atypical for FASI. Spinal MRI also demonstrated several lesions, which mildly enhance after administration of gadolinium. Cerebrospinal fluid (CSF) examination revealed mild lymphocytic pleocytosis (18/μL), mildly elevated protein (0.53 g/L), normal glucose, and positive oligoclonal IgG bands. Extensive laboratory workup, including microbiological CSF studies, aquaporin-4-IgG, myelin-oligodendrocyte glycoprotein-IgG, autoimmune screening, and viral serology, was negative. The genetic study revealed a new mutation in the NF1 gene that was not previously reported. We intend to discuss the genetic and autoimmune mechanisms by which MS and NF1 appear to be related and draw attention to this association because a timely diagnosis of MS is important to prevent further disability in NF1 patients.

## Introduction

Neurofibromatosis type 1 (NF1) is an autosomal dominant disorder caused by NF1 gene mutations on chromosome 17 and is considered a neurocutaneous syndrome, given the skin and nervous system involvement. It is characterized by the presence of café-au-lait spots, Lisch nodules on the iris, axillary and inguinal freckles, osseous lesions, and increased susceptibility to tumor formation, namely neurofibromas and optic pathway gliomas [1]. Multiple sclerosis (MS) is a chronic autoimmune disease that causes progressive demyelination of the brain and spinal cord. Although rare, the association between both conditions is reported in the literature, and it could be genetically related [2-5]. It is important to make an early MS diagnosis since early treatment can prevent further neurological disability for NF1 patients.

## Case presentation

A 25-year-old man with a previous history of depression and scoliosis was referred to a neurology outpatient clinic due to gait impairment with imbalance, tremor of the upper limbs, slurred speech and urinary symptoms in the form of urinary urgency and incontinence. These symptoms started a year earlier and had a progressive course. Reviewing the clinical history, the patient has an extensive plexiform neurofibroma of the right upper eyelid that underwent two surgeries, the first at the age of three and the last at the age of 24. When asked about family history, he mentioned that his father and sister have café-au-lait spots but no other associated symptoms.

**Figure 1:**
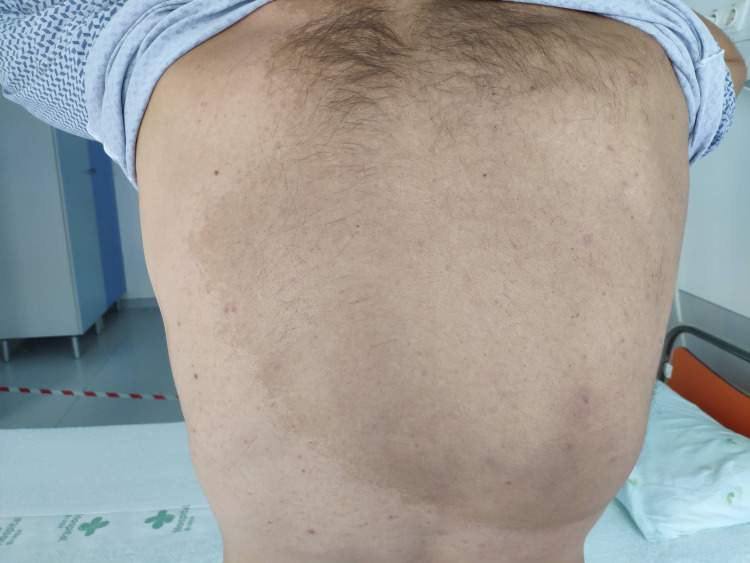
The largest café-au-lait spot of our patient in the torso.

The neurological examination showed right ptosis, bilateral horizontal and vertical nystagmus, mild dysarthria, mild quadriparesis (MRC 4/5), generalized hyperreflexia with ankle clonus, bilateral Tromner-Hoffmann and Babinski signs, postural and kinetic upper limb tremor, proprioceptive errors in both halluces, bilateral appendicular dysmetria of all limbs, titubation and severe gait ataxia. He also had multiple café-au-lait spots scattered throughout the trunk, the largest one located on the torso, extending from the shoulder to the lower edge of the ribs (Figure 1).

**Figure 2:**
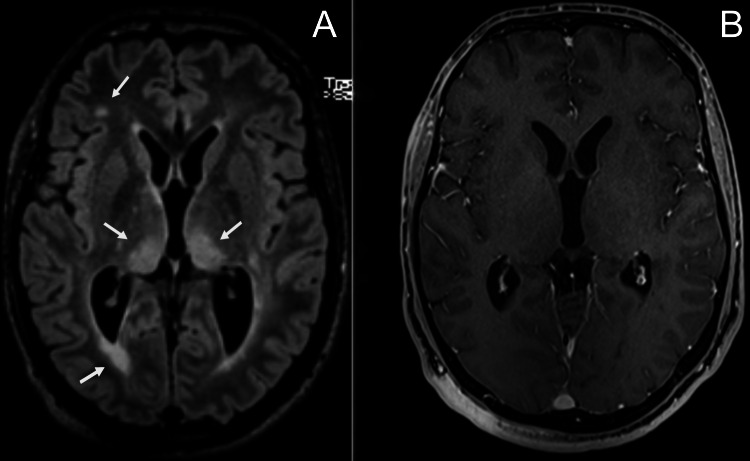
Brain magnetic resonance imaging, axial view. (A) Fluid-attenuated inversion recovery sequence (FLAIR) demonstrates several hyperintense lesions involving the deep white matter (mostly periventricular), the subcortical white matter in the right frontal lobe and both thalami (arrows); (B) they show no enhancement after administration of gadolinium on a fat-saturated T1-weighted volumetric interpolated breath-hold examination (VIBE) sequence. Their morphology and location are typical of focal areas of signal intensity (FASI) in the setting of neurofibromatosis type 1.

**Figure 3:**
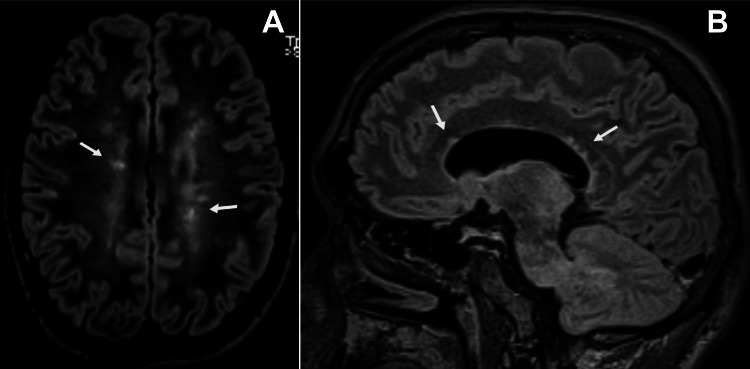
Brain magnetic resonance imaging, axial (A) and sagittal (B) view. (A) On an axial fluid-attenuated inversion recovery (FLAIR) sequence, several hyperintense lesions in the periventricular white matter can be depicted (arrows); they have an oval morphology, are perpendicular to the ventricles and involve the corpus callosum (this can be better appreciated on the sagittal view of a FLAIR sequence – B). They show no enhancement after administration of gadolinium (not shown).

**Figure 4:**
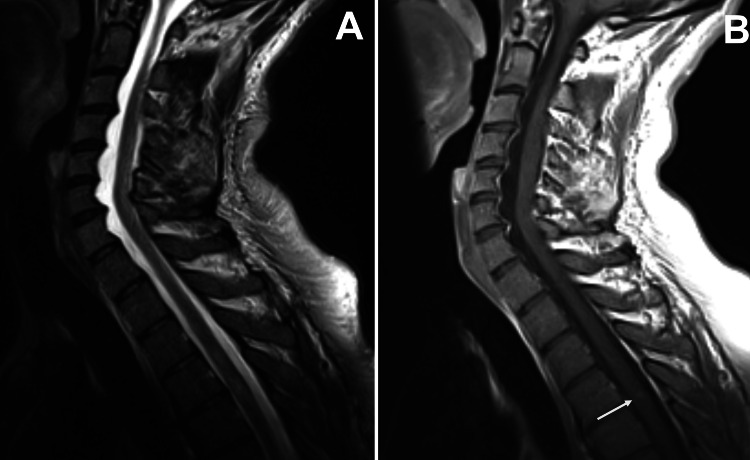
Spinal cord magnetic resonance imaging. (A) On a sagittal view of a T2-weighted turbo spin echo sequence, several hyperintense lesions involving the spinal cord can be observed; (B) one of them mildly enhances after administration of gadolinium on a sagittal view of a T1-weighted turbo spin echo sequence (arrow). Their location and morphology are atypical for areas of signal intensity (FASI) and could be related to a demyelinating process.

**Figure 5:**
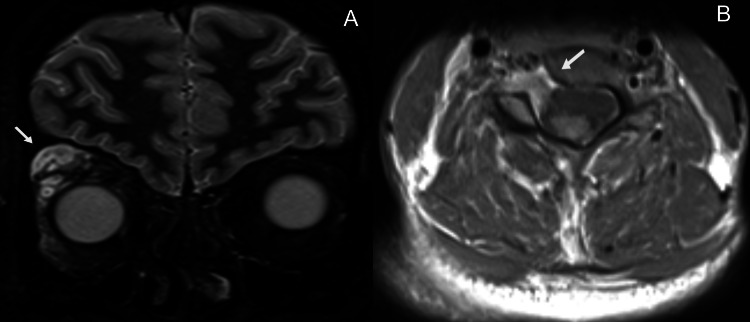
Brain (A) and cervical cord (B) magnetic resonance imaging. (A) On a coronal short tau inversion recovery (STIR) sequence, a globally hyperintense lobulated mass can be seen in the extraconal compartment of the right orbit, in keeping with a plexiform neurofibroma (arrow). (B) On an axial view of a T1-weighted turbo spin echo sequence, after administration of gadolinium, an enhancing mass in the right foramen of a cervical vertebrae can be depicted, suggesting a peripheral neurofibroma (arrow).

Brain magnetic resonance imaging (MRI) revealed several hyperintense lesions on fluid-attenuated inversion recovery (FLAIR) imaging involving the deep white matter, the subcortical white matter in the right frontal lobe and both thalami, that showed no enhancement on T1-weighted imaging (T1WI) after administration of gadolinium (Figures 2A-2B); their morphology and location were typical of focal areas of signal intensity (FASI) in the setting of NF1. Moreover, there were several hyperintense oval lesions on FLAIR in the periventricular white matter, perpendicular to the ventricles and involving the corpus callosum (Figures 3A-3B), as well as several hyperintense lesions on T2-weighted imaging involving the spinal cord, with mild enhancement on T1WI after administration of gadolinium (Figures 4A-4B); their location and morphology were atypical for FASI and were most likely related to a demyelinating process. A globally lobulated mass in the right orbit was also visible, in keeping with a plexiform neurofibroma (Figure 5A), and an enhancing mass in the right foramen of a cervical vertebra, suggesting a peripheral neurofibroma (Figure 5B).

Cerebrospinal fluid (CSF) examination revealed mild lymphocytic pleocytosis (18/μL), mildly elevated protein (0.53 g/L), normal glucose, and positive oligoclonal IgG bands. Extensive laboratory workup, including microbiological CSF studies, aquaporin-4-IgG, myelin-oligodendrocyte glycoprotein-IgG, B12 vitamin and folic acid, autoimmune screening (including ESR, ANA, ANCA, ENA panel, ACE, thyroid hormones and antithyroid antibodies, anti-dsDNA), and viral serology, was negative.

The genetic study revealed the following heterozygous mutation in the NF1 gene, c.888+1G>T. Although this genetic variant has not been published in the literature, other single nucleotide substitutions (G>A and G>C) in the same position have already been described and considered pathogenic due to splice donor site [6,7].

Ophthalmologic examination identified a bilateral reduction in visual acuity, temporal pallor of the optic disc, and a central scotoma on the right eye, without Lisch nodules. Moreover, the patient is being followed by Dermatology, Orthopedics, Physical and Rehabilitation Medicine, and Genetics. Currently, he is undergoing a rehabilitation program and awaiting approval to start ocrelizumab.

## Discussion

Our patient fulfilled diagnostic criteria for NF1 [8], further confirmed by the genetic study, and for MS [9], specifically the primary progressive form (PPMS). This association, although rare, is already described in the literature, with PPMS being the most frequent form of MS reported in NF1 patients, although all clinical forms can be found [4,5,10]. To our knowledge, this is the first Portuguese patient with NF1 and MS reported in the literature.

Recent studies confirmed a higher risk of MS among patients with NF1 [1,11]. One of the postulated theories for this association is related to a mutation in the oligodendrocyte myelin glycoprotein (OMG) gene that is embedded within intron 27b of the NF1 gene, encoded on chromosome 17q11.2. OMG is essential for myelination and could be dysfunctional in patients with NF1, leading to demyelination in MS-susceptible patients [10-15]. However, given that not all patients with PPMS have shown this mutation and that it was found in healthy controls, this cannot be the only explanation for this association [4,10,11]. Another hypothesis is related to neurofibromin, a product of the NF1 gene encoded in the same chromosome as OMG and highly expressed in myelin-forming oligodendrocytes [11]. Neurofibromin is a tumor suppressor protein that acts through the inactivation of p21 Ras protein. A mutation in the NF1 gene can lead to activation of the p21 Ras protein and consequent excessive cell proliferation, leading to the appearance of tumors. Therefore, the lack of this suppressor protein can lead to overactivity of other systems, such as the immune system, resulting in an autoimmune response against central nervous system (CNS) myelin [5,10]. Finally, in addition to these genetic hypotheses, it is also postulated that NF1 is a proliferative disease involving Schwann cells, and abnormal exposure to peripheral myelin can trigger an autoimmune response against the same antigens expressed in CNS myelin [5].

Both NF1 and MS can show abnormalities in brain and spinal imaging, with some features helping to differentiate both lesions [16,17]. Brain MRI of most patients with NF1 demonstrates focal areas of high signal intensity on T2-weighted sequences, which are called FASI. These areas resemble MS lesions, although some differences can be highlighted: first, FASI occur mainly in the basal ganglia, brainstem, and cerebellum, and do not enhance after gadolinium administration; in contrast, MS lesions are typically located in the corpus callosum, periventricular, or cortical/juxtacortical areas, show an oval morphology, and can enhance after gadolinium (i.e. the active lesions); second, MS lesions can change over time, varying in size, number, location, and pattern of enhancement (depending on its activity), while NF lesions (i.e. FASI) grow slowly typically to the age of 10 and stabilize after that; finally, NF lesions tend to be larger than MS lesions and are not surrounded by edema or mass effect [18].

NF1 can present with several neurological manifestations, such as epilepsy, intellectual disability, vision loss, macrocephaly, bowel/bladder dysfunction, and/or motor weakness. However, the demyelinating lesions of our patient do not fit this disease and we intend to draw attention to the fact that not all neurological symptoms can be attributed to NF1 [4,13-15].

## Conclusions

We report a Portuguese patient with NF1 and MS, namely a primary progressive form, and describe a new NF1 mutation that was not previously reported. Both diseases appear to be related, possibly by genetic and autoimmune mechanisms. Some neurological manifestations may be similar between them, for example, vision loss due to optic gliomas in NF1 and optic neuritis in MS or bladder/bowel dysfunction as a result of spinal cord lesions in MS due to spinal root tumors in NF1. Thereby, NF1 patients should be regularly monitored and every new complaint or unusual clinical manifestation must be investigated to promptly diagnose and treat other comorbidities, preventing further disability.
